# Distributed Supervision Model for Enterprise Data Asset Trading Based on Blockchain Multi-Channel in Industry Alliance

**DOI:** 10.3390/s22207842

**Published:** 2022-10-15

**Authors:** Jianxiong Zhang, Bing Guo, Xuefeng Ding, Dasha Hu, Yuming Jiang

**Affiliations:** 1 College of Computer Science, Sichuan University, Chengdu 610065, China; 2 Big Data Analysis and Fusion Application Technology Engineering Laboratory of Sichuan Province, Chengdu 610065, China

**Keywords:** data asset, data trading, distributed supervision, blockchain, multi-channel

## Abstract

Compared with traditional physical commodities, data are intangible and easy to leak, and the related trading process has problems, such as complex participating roles, lengthy information flow, poor supervisory coverage and difficult information traceability. To handle these problems, we construct a distributed supervision model for data trading based on blockchain, and conduct multi-party hierarchical and multi-dimensional supervision of the whole process of data trading through collaborative supervision before the event, at present and after the event. First, the characteristics of information flow in the data trading process are analyzed, and the main subject and key supervision information in the data trading process are sorted out and refined. Secondly, combined with the actual business process of data trading supervision, a multi-channel structure of distributed supervision is proposed by adopting an access–verification–traceability strategy. Finally, under the logical framework of the supervision model, the on-chain hierarchical structure and the data hybrid storage method of “on-chain + off-chain” are designed, and multi-supervisor-oriented hierarchical supervision and post-event traceability are realized through smart contracts. The results show that the constructed blockchain-based distributed supervision model of data trading can effectively isolate and protect sensitive and private information between data trading, so as to realize the whole process, multi-subject and differentiated supervision of key information of data trading, and provide an effective and feasible method for the controllable and safe supervision of data trading.

## 1. Introduction

With the accelerated pace of digital transformation and upgrading of enterprises, data, as a new factor of production, have gradually become the core resource for enterprise competitiveness and soft power, and an important asset of enterprises [1,2]. Data assets refer to data resources that are owned or controlled by various entities and can generate value for the owner and are recorded physically or electronically. The capitalization of data resources provides the basis for data trading between enterprises. Data trading is a key link in the market-oriented configuration of data elements, which can accelerate the cultivation of the data element market, promote the effective flow of data assets, and tap and play the value of data assets. It is one of the important means to realize the value increment of data. However, most traditional data trading methods are dominated by a centralized management mode. With the rapid growth in the scale and quantity of data asset trading, due to weak or even a lack of supervision in the trading process, the sensitivity, privacy and security issues of data resources and data trading have become increasingly prominent [3,4], which has become the main obstacle for enterprises to participate in data trading activities.

Firstly, traditional database storage itself has certain risks. In addition, the supervision of enterprise data asset trading involves laws, regulations and economic penalties, which may result in tampering with trading information to avoid penalties [5,6,7,8]. Secondly, as a kind of digital resource, data assets are obviously different from traditional physical commodities, which are intangible, easy to copy, and difficult to estimate, as well as undergo fast growth [9,10]. Moreover, the entire life cycle of data asset trading is long, including data service demand, data resource provision, data processing, data processing result feedback and other links. Different trading links are usually used between different enterprises, and the data trading information is also distributed in storage and maintenance, which leads to the difficulty of data trading supervision and other problems. Finally, on the one hand, the information of each link of data trading should be shared to facilitate supervision. On the other hand, sensitive and private information in each link needs to be protected, leading to a contradiction between supervision and privacy [11,12]. Therefore, how to solve the two major issues of privacy and supervision, denial and traceability, has become an urgent problem to realize enterprise data asset trading.

The emergence of blockchain technology has brought feasible solutions for standardized data management, which has been widely used in data asset trading, data ownership protection, product traceability and other aspects. Silvestre et al. [13] studied innovative applications in the field of power systems and the development of blockchain in ancillary services and electricity markets around blockchain technology. Mehrdokht et al. [14] discussed the application of blockchain technology in supply chain, logistics and transportation management for the four pain points of the supply chain: technology, trust, trade and traceability. Casino et al. [15] applied blockchain technology to the traceability of food supply chains. Due to its data security, transparency, non-tampering and traceability characteristics [16,17], blockchain technology has been applied in different fields to solve related problems [18,19,20,21,22]. However, there are relatively few studies on the application of blockchain technology in the supervision of enterprise data asset trading.

Aiming to solve the above problems, this paper comprehensively analyzes the business process of data trading and its supervision characteristics, sorts out and extracts the main subject and key supervision information of each business link of data trading, and maps them to the blockchain. According to the actual supervision requirements of data trading, we adopt the access–verification–traceability strategy, and combine it with blockchain multi-channel technology to build a distributed supervision model of data trading, so as to realize the isolation and protection of sensitive and private information of each data trade in the whole process of supervision. On the basis of the supervision model, its on-chain hierarchical structure, the hybrid storage mode of on-chain and off-chain, the on-chain supervision process and the trading information traceability process are proposed, and a supervision smart contract is used to realize the hierarchical supervision and ex-post traceability for multiple supervisors. Finally, the proposed model and blockchain network are verified and analyzed by simulating multi-node deployment. This provides important ideas and methods to solve the contradictions between regulation and privacy, denial and traceability in the study of data trading supervision, and provides more reliable and secure data asset trading services for industry alliance enterprises.

## 2. Related Work

Data trading: In recent years, blockchain-based data trading models have attracted more and more attention from scholars. Jung et al. [23] proposed a set of accountability protocols named AccountTrade to blame dishonest consumers in data trading to achieve a secure big data trading environment. However, this work assumes that the broker is trustworthy, which may lead to privacy leaks and risks in practical application scenarios. Dai et al. [24] proposed a data trading ecosystem based on blockchain and Software Guard Extensions, in which neither the data broker nor the buyer can access the seller’s raw data, but only the data analysis results. Ramachandran et al. [25] propose a novel distributed publish–subscribe broker that stores data in an immutable ledger through blockchain technology, facilitating the transparency of participant interactions and data status. However, the data are stored on the blockchain in plaintext, which is not suitable for sensitive data. Wang et al. [26] proposed a data trading scheme based on the Bitcoin system. In their scheme, the digital content is encrypted by a symmetric key, which is then encrypted by an RSA scheme. Dib et al. [27] propose a novel blockchain-based framework in which the service provider will not own copies of the data, but utilize models on top of the data, protecting both the user’s data and the service provider’s model. However, they assume that the data owners in their system are honest, which is not suitable for most data trading scenarios. Zhao et al. [28] proposed a blockchain-based data fair trading protocol that integrates technologies such as ring signature and a double-authentication-preventing signature to enhance the privacy, availability, and fairness of data trading. Niu et al. [29] proposed a security mechanism for the personal data market, which achieves authenticity and privacy protection by using homomorphic encryption and identity-based signatures. Kang et al. [30] proposed a P2P data trading strategy for vehicular computing and networks. It uses consortium blockchain and smart contracts to achieve secure data caching, effectively preventing unauthorized second-hand data sharing. Li et al. [31] introduced a decentralized fair data trading framework, and integrated technologies such as homomorphic encryption, smart contracts and double-authentication-preventing signatures to improve data availability and achieve fair data trading.

Security supervision: Researchers have investigated the application of blockchain technology in security supervision from different perspectives. Baralla et al. [32] built a blockchain system to manage and track the food supply chain, which guarantees transparency, efficiency and trustworthiness throughout the process through the use of smart contracts. Wang et al. [33] proposed a framework using Hyperledger smart contracts to track and trace the workflow of agricultural supply chains and improve the integrity, reliability and security of trading records. Based on the analysis of the traditional Chinese medicine supply chain, Li et al. [34] constructed a TCM quality and safety traceability system based on blockchain technology to solve important supply chain traceability problems. Yong et al. [35] proposed an intelligent system based on blockchain and machine learning technology for issues such as vaccine expiration and vaccine fraud in the vaccine supply chain. On the basis of analyzing the key information of each link of the rice supply chain, Wang et al. [36] constructed a blockchain-based rice supply chain information supervision model and adopted a hierarchical data encryption storage model to ensure the security and privacy of data in the process of circulation and storage. Aiming to solve the problem that model updates in FL are easily tampered by malicious agents, Wei et al. [37] proposed an efficient chameleon hash scheme for secure federation learning in Industrial Internet of Things. To address the security of the key used for encryption and decryption of industrial IoT data, Yu et al. [38] proposed a blockchain-based threshold encryption protection scheme for IIOT data, which uses the private key of the edge gateway to protect the symmetric key. Tan et al. [39] proposed a blockchain-based general access control framework for green smart devices (GSD), which reduces the complexity of user access and control of heterogeneous GSDs by leveraging the decentralized and non-tampering features of blockchain. Górski [40] discussed the pattern of smart contract design and implementation, introduces the advantages of reusability and security in detail, and demonstrates it. In order to improve the fairness and security of credit evaluations in the e-commerce system, Xiao et al. [41] proposed an e-commerce transaction system based on blockchain, which includes a reputation evaluation scheme based on multi-criteria decision making and an incentive mechanism based on reputation value.

The above research combines the application of Internet of Things technology and blockchain, with much work carried out for the construction of the data trading platform, physical commodity safety supervision and traceability. However, there are few studies on the security supervision and traceability of data trading, which does not satisfy the actual trading requirements of data, a special commodity, in application scenarios.

To address this shortcoming, this study analyzes the particularity of data commodities and their trading as well as the supervision information in each link of the trade, and provides a classification table of key regulatory information in the main links. Combined with the Hyperledger Fabric channel technology, we propose a distributed supervision model for data trading based on multi-channel technology, which utilizes the natural isolation of channels to meet the requirements of efficient supervision while protecting the sensitive and private information of trading subjects in each trading link. In addition, on the basis of forward supervision, the reverse supervision of the trading process is realized, which solves the problems of differentiated supervision and penetrating supervision. In summary, this study helps to optimize the supervision breadth and depth of the supervisor in data trading, and provides a feasible and effective solution for the security supervision of data trading in the future.

## 3. Analysis of Key Supervision Information and Problems in Data Trading Process

### 3.1. Analysis of Data Trading Process and Key Supervision Information

**Figure 1:**
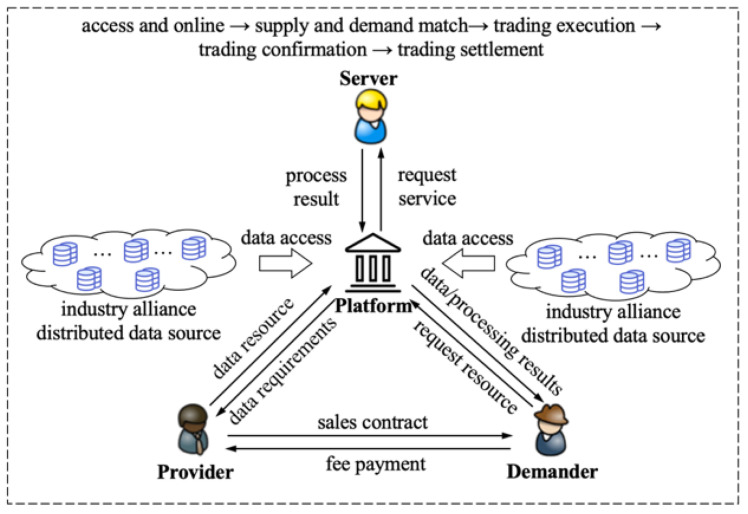
Schematic diagram of the whole process of data asset trading. Data access and online includes compliance review, standardization check, data catalog upload, dataset or data service demonstration sample upload.

The whole process of data trading can be divided into five typical links: access and online, supply and demand match, trading implementation, trading settlement and data service, which logically includes three types of trading entities of data service demanders, providers and servers, and platforms and supervisors. The whole process of trading data assets is shown in Figure 1.

(1) Demander: This refers to the party that initiates the data service request and is the subject that purchases the data service. The demander requests data resources or data services from the provider or server through the platform.

(2) Provider: This provides the original data resources and the owner of the data assets. After receiving the data resource request through the platform, the provider will directly provide the data resource to the demander or provide it to the server for further processing, and endow it with the ownership or right to use the data resource.

(3) Server: This has a mature model or algorithm that exhibits an excellent performance, and is responsible for processing the data resources of the provider and providing data services at the request of the demander or the authorization of the provider through the platform.

(4) Platform: This is a comprehensive information platform for the business activities of three types of trading entities and supervisors. The first case is that the demander requests data resources and feedback from the provider through the platform. The second case is that the demander requests data services and feedback from the server through the platform, and the server can request data resource authorization from the provider through the platform when there is no data resource. Furthermore, the platform can also directly provide conventional data services to the demander.

(5) Supervisor: This refers to the party that supervises the three types of trading entities, platform and the whole process of data asset trading, mainly including third-party supervision authorities, platform and representatives of alliance members other than trading parties.

Furthermore, due to the intangibility and easy replication of data commodities, their trading is also different from traditional trading activities, with the following characteristics:

(1) Data leakage risk: Data are different from physical commodities and can be easily copied. Once leaked, they are difficult to recover.

(2) Invisibility: Due to the intangibility of trading objects, the virtualization of trading supervision channels and the concealment of trading forms, the trading process is not easy to monitor and track, and it is easy to deny.

(3) Flexibility: Compared with the trading of physical commodities, the trading subject, time and method of data trading have great flexibility and freedom.

In conclusion, the data trading process involves many subjects and complex links, and has the characteristics of easy leakage, intangibility and flexibility. Therefore, the supervision of data trading is also more complicated than ordinary physical commodity trading. In the actual supervision process, not all trading information is used for supervision. In the process of data trading, sensitive and private information (such as content, price, etc.) of enterprises and individuals is also involved. Among them, sensitive information such as prices cannot be completely transparent and open, resulting in conflicts between supervision and the protection of sensitive and private information. Additionally, each link of data trading is usually carried out between different enterprise entities, and the privacy information of each link is difficult to effectively protect. It is difficult to achieve effective supervision, resulting in frequent safety problems in data trading, which hinders the flow of data assets.

In this paper, a whole-process, multi-party and multi-level strategy is adopted to comprehensively supervise data trading, in which the supervision party is composed of supervision agencies, a platform, and representatives of alliance members unrelated to the trade. These supervisors implement collaborative supervision and classified and hierarchical controllable multi-party collaborative supervision before the event, at present and after the event over the entire process of data trading through hierarchical responsibilities. Among them, pre-supervision is access verification, referred to as access. In-process supervision is verification comparison, referred to as verification. Post-event supervision is retrospective audit, referred to as traceability. In the whole process of data trading, all data trading will be supervised in the form of “A-V-T (access-verification-traceability)”, but not all information generated in the whole process will be used for supervision. The supervision of data trading is mainly used for compliance verification, comparison, recording and verification of the behavior and information of key links in the whole process of the trading. Obviously, the behavior and information of key links in the whole process of the trading may involve sensitive and private information, which needs to be classified and controlled and effectively protected.

Note: Sensitive and private information and supervisory roles should be classified and graded, but this paper focuses on the data trading supervision mode and method, and does not involve system development. Therefore, the classification of sensitive and private information and supervisory roles are not discussed here.

In view of the above problems and requirements, on the basis of ensuring the supervision and realizing classified and hierarchical controllable multi-party collaborative supervision before the event, at present and after the event of data trading, in order to improve the supervision efficiency and ensure the effective protection of private information in each link, this paper extracts the key information of each link, and divides it into public supervision information and private supervision information (as shown in Table 1), which are regarded as the key information for data trading supervision.

The detailed information classification can further optimize the process of the data trading supervision and can be used as the basis for establishing a whole-process supervision model for data trading.

### 3.2. Problems in Traditional Data Trading Supervision

Data trading supervision is conducted to take supervision agencies, platform and enterprise representatives as the main supervision subjects, and uses legal means and related technologies to supervise and manage data trading behavior, so as to realize the safe and orderly conduct of data trading.

The objects of supervision mainly include four aspects: trading subject, trading object, trading process and trading platform. In accordance with the trading rules and regulations, the supervisor uses trading behavior and information records to conduct compliance A-V-T (access–verification–retrospective) supervision on the trading process and results, and analyze the possible violations of laws and regulations in the trading through market data. At present, the main problems in enterprise data asset trading are as follows:

(1) Privacy of trading: The supervision of data asset trading covers the whole process of data trading, which includes multiple links. Trading information may include original data and derived data of enterprise organization information, involving the personal privacy and business secrets. Different trading links are usually carried out between different trading entities, so the trading information of each link has a certain degree of privacy. For example, if the data asset information between the platform and the provider is leaked, data may be maliciously collected and resold, resulting in data black production.

(2) Privacy of the supervisor: The supervision of data asset trading is omnibearing and full-coverage supervision, which involves the hierarchical cross-supervision of multiple supervisors at the same time, including third parties and members of alliance companies outside of the trade. The information of various supervisors is private and needs to be effectively protected.

(3) Tampering of trading data: The traditional supervision mode stores trading information through the enterprise’s local database, resulting in unclear data trading requirements, a poor data flow and lengthy information flow between enterprises. Additionally, there may be dishonest companies tampering with trading information in various links; these problems reduce the credibility of the supervision results. Moreover, the traditional data trading supervision mode requires a lot of repeated verification and inspection of trading information in each link, which leads to high time costs, a lengthy information flow and low supervision efficiency.

(4) Credible traceability is difficult and easy to deny. Due to the lack of reliable evidence or the difficulty in locating the responsible subject, follow-up accountability is unsustainable, and the rights and interests of all trading subjects have been seriously infringed. In the link of data trading, the difficulty of mutual trust increases the cooperation cost between participants. Meanwhile, the centralized operation mode tends to make supervision information opaque, leading to a low reliability of supervision and traceability information.

To sum up, the above-mentioned potential problems and hidden dangers are essentially the two main problems of privacy and supervision, denial and traceability, which seriously challenge the security and healthy development of data trading, resulting in the disorder and increased risk of the trading market. If this goes on for a long time, it will cause serious damage to the entire data trading industry and even the public interests of society and countries. The introduction of blockchain will help strengthen non-tampering and non-repudiation, enhance controllable transparency, rebuild consensus and trust, improve the traceability of trading information in all links, strengthen trading supervision, and avoid risks. This effectively solves the two major issues of privacy and supervision, denial and traceability, and improves the quality and efficiency of data trading.

## 4. A Distributed Supervision Model for Enterprise Data Asset Trading in Industry Alliance

The whole process of enterprise data asset trading covers multiple links of trading activities, involving multiple enterprise entities, and enterprise data assets are distributed and stored in the enterprise local database, which makes data trading difficult to supervise. At the same time, supervision is usually completed in parallel by the division of labor among multiple supervisory roles. The supervisor obtains the trading information of each link from each trading subject (demander, provider, and server) and the platform, and implements distributed parallel supervision through the analysis of the main links. In the supervision, the sensitive and privacy information of trading activities is effectively isolated and protected to provide security guarantee for trading activities.

In the traditional centralized trading mode, since data trading involves the interests of all parties, there is a risk of trading information being tampered, and the opaqueness of trading information will also affect the trust of both parties. Furthermore, data resources also have problems such as privacy leakage and resale caused by malicious collection, which makes many companies question or worry about the privacy and security of data trading activities. The blockchain technology has the characteristics of decentralization, anonymity and immutability, which provides a new method of data trading supervision. Meanwhile, if there is a lack of supervision of trading activities, even if the immutability of the blockchain guarantees that trading can be traced, it still cannot change the established facts. Therefore, the distributed whole-process supervision mechanism based on blockchain is introduced into the data asset trading activity supervision model, which can supervise the data asset trading activity in the enterprise dynamic alliance more effectively.

### 4.1. Blockchain and Fabric Channel Technology

#### 4.1.1. Blockchain Technology

**Figure 2:**
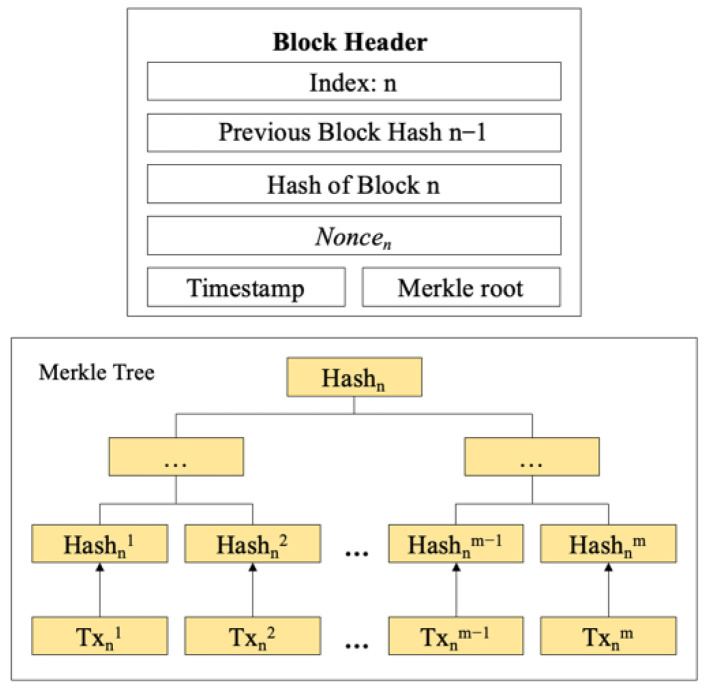
Basic structure of block.

The essence of blockchain is a decentralized distributed ledger system [42]. The verification and storage of transactions are completed by the cooperation of the whole chain nodes, and the changes of the state of the ledger are realized by running a consensus algorithm between nodes. The storage structure of the blockchain is shown in Figure 2, and a block consists of a block header and a block body. The hash value of the previous block is stored in the block header [43], and the detailed transaction information is stored in the block body. The traceability and security reliability of information are enhanced [44].

#### 4.1.2. Hyperledger Fabric Multi-Channel Technology

**Figure 3:**
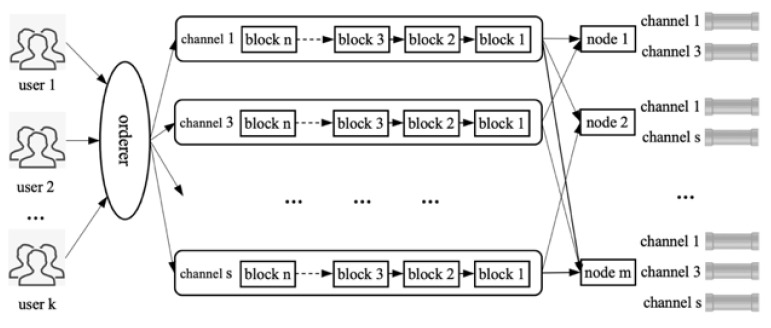
Hyperledger Fabric multi-channel technology.

Hyperledger Fabric [45,46] is an enterprise-level, open-source, permissioned blockchain platform with features such as privacy and permission, and can creates channels and supports a variety of hot-pluggable methods. In the Fabric consortium chain, relevant transaction nodes create corresponding channels to shield the information in the channel to the outside world to ensure the privacy of transaction activities in the channel. Multiple channels can be created in the Fabric consortium chain [47], and a node can be authorized by the CA certificate to participate in multi-channel transaction activities at the same time. Its organizational structure and operation mode are shown in Figure 3. Fabric channels are divided into system channels and application channels. System channels are created and recorded with the startup of Fabric, which are used to create and manage application channels and serve the entire consortium chain network. The application channel is created by the relevant transaction nodes according to their own needs, and formulates corresponding strategies (roles, permissions, sorting, etc.) to carry out transaction activities.

### 4.2. Distributed Supervision Overall Architecture for Enterprise Data Asset

Data trading can greatly promote the flow and sharing of data and activate the value of data elements. Strengthening the supervision of the whole process of data trading is one of the important measures to ensure the smooth progress of data trading activities. The supervision of data trading involves multiple trading links, while the traditional data trading supervision system only stores trading information in the enterprise centralized database. The blockchain uses distributed ledgers to back up the supervision information of the data trading in the centralized database, which can record and reversely trace the source of trading activities, and solve the technical problems of data trading supervision between enterprises caused by consensus and trust. Blockchain supervision nodes query the data ledger through indexes such as block index or transaction hash, but cannot achieve differentiated sharing of data on the chain. The blockchain storage structure can ensure that block data cannot be tampered with and cannot be deleted, which can be permanently traced.

**Figure 4:**
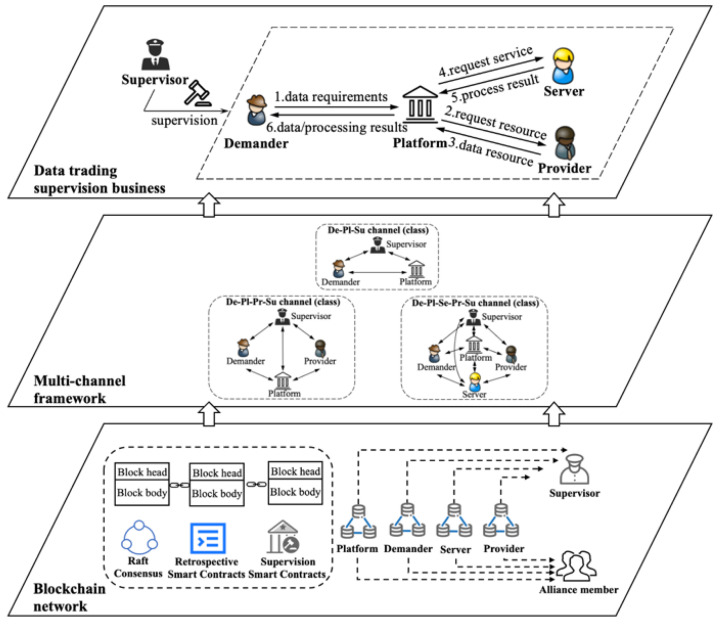
Blockchain-based distributed supervision model for data trading.

In response to the above problems, according to the current situation and characteristics of data trading, the strategy of off-chain distributed storage and centralized trading, as well as on-chain distributed supervision, is adopted. That is to say, the data sources of data trading are still distributed and stored in the databases of each enterprise, and the trading activities of each participant are all centralized on the unified platform under the chain. The data trading supervision adopts a multi-party and multi-level distributed supervision mode, and completes the whole process of data trading supervision tasks on the chain. Therefore, this paper proposes a distributed supervision model for data trading based on blockchain. As shown in Figure 4, the model includes the access–verification–traceability collaborative supervision of the whole process of data trading. Through the collaborative supervision before the event, at present and after the event of data trading activities, multi-party, multi-level and multi-dimensional supervision is realized to ensure that the whole process of data trading is traceable and cannot be tampered with.

Based on the distributed supervision model (as shown in Figure 4), a consortium chain business framework with three types of data trading supervision channels is proposed, which includes an industry alliance data trading supervision chain and three types of data trading supervision channels. Among them, the three types of data trading supervision channels belong to the application channel; each type of supervision channel can include many specific supervision channels, and each data trading under the chain creates a one-to-one corresponding supervision channel on the chain. According to the specific business differences of the three types of data trading, three types of data trading supervision channels are created corresponding to them. De-Pl-Su (Demander–Platform–Supervisor) deals with the situation where the platform side directly provides regular data services to the demander. De-Pl-Pr-Su (Demander–Platform–Provider–Supervisor) deals with the situation where the demander requests data resources and feedback from the provider through the platform. De-Pl-Se-Pr-Su (Demander–Platform–Server–Provider–Supervisor) deals with the situation where the demander requests data services and feedback from the server through the platform. When there is no data resource, the server can request data resource authorization from the provider through the platform. Moreover, the industry alliance data trading supervision chain also has a system channel, which is responsible for classifying and managing all channels on the alliance chain. On the Fabric, multi-channel technology is used to create three types of supervision block channels, De-Pl-Su trading, De-Pl-Pr-Su trading and De-Pl-Se-Pr-Su trading, which are used for the supervision of different types of data trading. According to the A-V-T (access–verification–traceability) collaborative supervision requirements, the key information of the main links of the off-chain data trading is uploaded to the corresponding supervision channel on the chain. On this basis, traceability chains oriented to traceability requirements and controllable supervision have been established, in which the key information of each data trade is stored, accessed and traced in isolation from each other through the channel, protecting privacy information, and realizing classified and hierarchical controllable multi-party collaborative supervision before the event, at present and after the event.

In order to ensure the healthy, sustainable and stable development of the blockchain supervision network, data trading entities first need to obtain authorization documents from the supervision authorities and industry alliance licenses before they can enter the data trading supervision alliance chain. The natural isolation of the channel is used to ensure the confidentiality, privacy and security of information between different data trades.

### 4.3. Blockchain Multi-Channel Structure for Distributed Supervision

**Figure 5:**
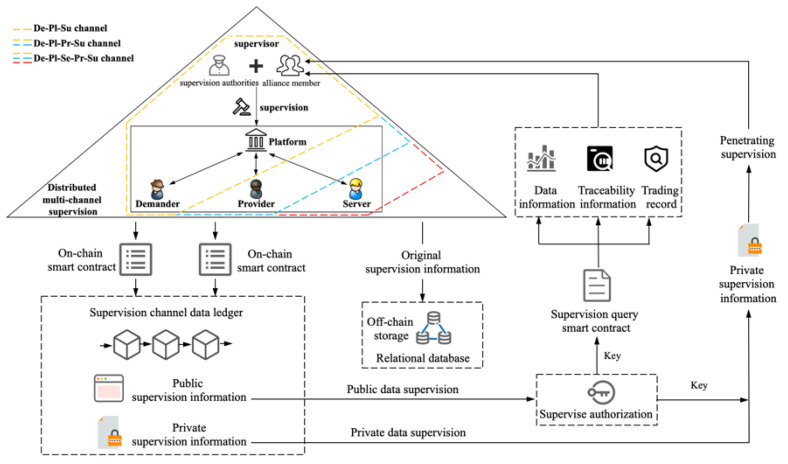
On-chain supervision process of data trading.

Various data asset trading activities exist and are carried out in industry. On the basis of analyzing the characteristics of data trading, a multi-channel structure of a blockchain-based distributed supervision model was constructed based on channel technology, as shown in Figure 5. Fabric multi-channel technology isolates various activities logically, and provides new technology for orderly organization of various trading activities and no leakage of sensitive and private trading information. First, independent channels (such as De-Pl-Su, De-Pl-Pr-Su, and De-Pl-Se-Pr-Su trading supervision channels) are set up for each trading activity, and each supervisor node in the supervision channel chooses to join different channels according to actual requirements, which logically guarantees the effective isolation and security protection of each trading information. Secondly, in the model, the platform side can allocate its own blockchain nodes and the blockchain nodes of the demander, server and provider. Three types of supervision channel (such as De-Pl-Su, De-Pl-Pr-Su, and De-Pl-Se-Pr-Su) peer nodes were set up to coordinately supervise each trading activity in the three types of data trading, and were used to upload the trading information of the three types of data trading activities to the chain, which ensures the isolation and privacy protection of individual trading activity information. Finally, the blockchain also needs to set up an orderer node to uniformly sort and batch all trading.

The on-chain supervision process of data trading is shown in Figure 5. The supervision information is recorded from the trading supervision channels until the data service is provided according to the data trading link. Sensitive trading information is encrypted and stored by the trading supervision channel for authorized access, public information is transparently supervised by the supervision channel, and data trading information is managed and controlled by all the supervisors. Among them, the trading supervision channel can exchange information through the system channel of the alliance supervision chain. When the trading information is uploaded to the chain, the supervision smart contract is first called to perform pre-supervision to judge its regulatory, business and technical compliance (whether the content, ownership and data format comply with regulatory regulations). Then, the on-chain smart contract is called to record in the enterprise node and update the blockchain enterprise node database and block index records. Finally, the channel smart contract is triggered to upload the trading supervision information to the corresponding channel of the alliance supervision chain.

### 4.4. On-Chain and Off-Chain Hybrid Storage of Data Trading Supervision Information

**Figure 6:**
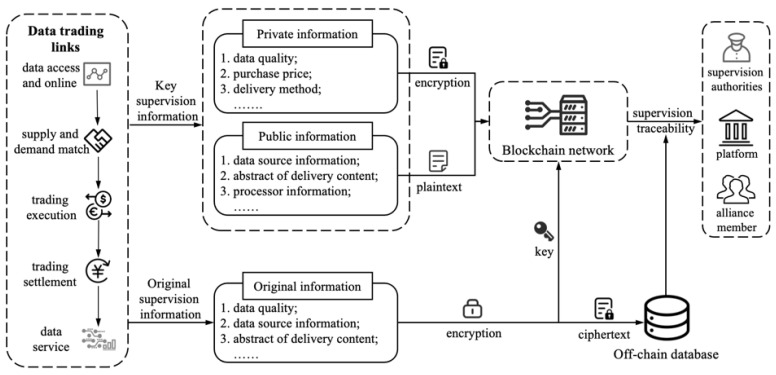
Data storage method for data trading supervision based on blockchain.

**Figure 7:**
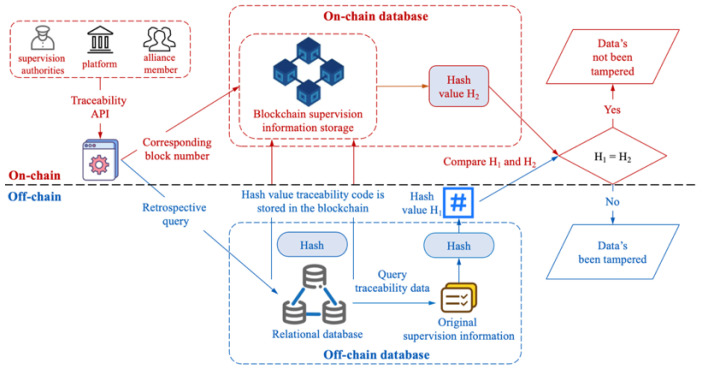
The verification for the traceability process of blockchain data trading supervision information.

In the process of data trading, the source data being traded are usually multi-source, heterogeneous, massive and so on. If all the source data and trading information of data trading are stored on the blockchain, the operation cost will be extremely high and the operation efficiency is extremely low. Meanwhile, data trading supervision only needs to collect the key information of the main links in the process of data trading (Table 1). Therefore, the proposed distributed supervision model adopts the hybrid storage method of the “on-chain + off-chain” database, in which the off-chain database is a database system for each enterprise to store source data and the platform to store all trading information in the whole process of data trading. Only key supervision information is stored on the blockchain, such as: data digests, timestamps, digital signatures, hash traceability codes and other information. The hybrid storage method of “on-chain + off-chain” cannot only ensure the security and credibility of the source data, but also improve the computing efficiency of the blockchain. The blockchain-based data trading supervision data storage method is shown in Figure 6. The on-chain algorithms for private and public trading supervision information are shown in Algorithm 1 and Algorithm 2, respectively. The symbols that will be used later in this section are shown in Table 2.
**Algorithm 1**: Smart contract for private supervision information on-chain**Input:**EnterPeer, $Key_{supchan}$, PriSupInfo   **Output:**txID, BlockNum 1 // Verify the validity of supervision channel authorization file 2 If val($Key_{supchan}$) 3 // Pre-chain supervision of trading information 4 if ((isTdTypeLegal(PriSupInfo) && isTdContentLegal(PriSupInfo)) 5   // Store the data trading information into the corresponding channel 6   if (wriSupChannel(EnterPeer, $Key_{supchan}$, PriSupInfo)) 7  return txID, BlockNum;

**Algorithm 2:** Smart contract for public supervision information on-chain**Input:**EnterPeer, PubSupInfo **Output:**txID, BlockNum 1 // Check the format and content of data trading information 2 if ((isTdTypeLegal(PubSupInfo) && isTdContentLegal(PubSupInfo)) 3 // Store the data transaction information in the blockchain public area 4 if (wriPubChannel(EnterPeer, PubSupInfo)) 5  return txID, BlockNum;

The hash value of the trading information stored in the blockchain is generated by the hash calculation according to the key information of the main links of the data trading. Once the source data or the above-mentioned key information in the off-chain database is tampered with; the trace code calculated using the hash value will change. If it is inconsistent with the corresponding trace code stored in the blockchain, the data have been tampered with. Figure 7 shows the traceability process of data trading information on the blockchain. The verification algorithm of data trading information traceability process is shown in Algorithm 3.

Taking the traceability query of data trading information as an example, the traceability information fields stored in the off-chain database of the platform include: ID, BatchNumber, TradingName, TradingContent, TradingNumber, Operator, TradingTime, and BlockNumber. Among them, ID is the unique identifier of the trading information, and BlockNumber is the block number of the hash value of the traceability information on the blockchain.
**Algorithm 3:** Verification algorithm of data trading information traceability processInput: SupPeer$,\;Key_{supcha}$$,\;Key_{oriinfo}$, TradingName   Output: Veriresult 1 // Query the original data trading information and its corresponding block number from the off-chain database 2 OriTradingInfo=queryTradingInfo(SupPeer$,\;Key_{oriinfo}$, TradingName); 3 // Compute the hash value of the original data trading information $4\;H_{1}=\mathrm{calcHash}(OriTradingInfo);$ 5 // Query the hash value in the blockchain based on the block number $6\;H_{2}=\mathrm{queryTradingInfo}(SupPeer$$,\;Key_{supchan}$, OriTradingInfo[7]); $7\;\mathrm{If}\;(H_{1}$$==H_{2})$ 8   return Veripass; 9 else 10   return Verifail;

### 4.5. On-Chain Layer Structure of Data Trading Supervision Model

**Figure 8:**
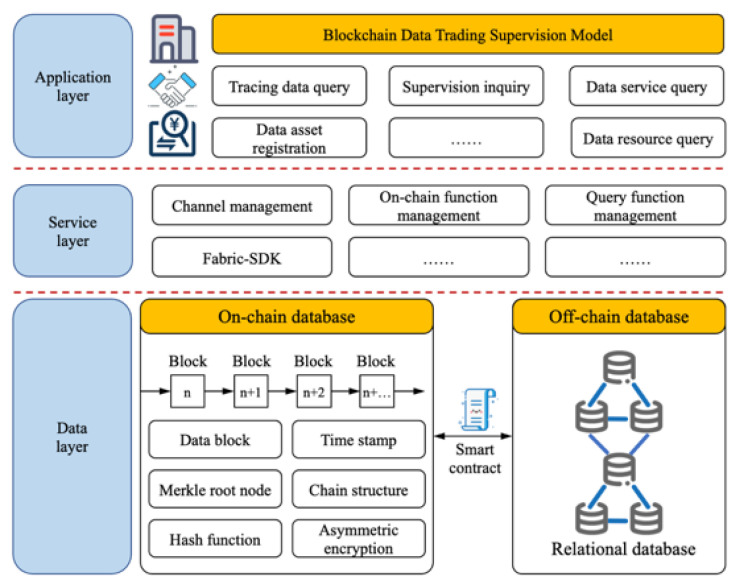
On-chain layer structure of data trading supervision model.

The on-chain layer structure of the proposed blockchain data trading supervision model adopts a classic three-layer blockchain structure, including data layer, service layer and application layer, as shown in Figure 8. The data layer adopts the data hybrid storage method of “on-chain + off-chain”. The service layer is responsible for the interaction between the application layer and the blockchain network. The application layer provides a variety of application functions for various users who participate in and supervise data trading.

(1) Application layer: The application layer mainly provides enterprise users with a data trading supervision and management window through mobile and PC applications. On the basis of the existing blockchain network, the application layer accesses the smart contracts installed on the peer nodes through the API interface provided by the underlying blockchain, and operates the ledger data. The objects of this layer mainly include supervisory agency, the platform and the representatives of the alliance members, that is, the representatives of enterprises unrelated to this trade. This layer is used to realize the business requirements of data trading activity traceability and data analysis.

(2) Service layer: The service layer is responsible for the interaction between the application layer and the blockchain network. It provides various APIs for the application layer, interacts with the blockchain network layer, and maps the logical operations of the application layer to the blockchain network. The service layer will also effectively manage the blockchain network layer. For example, the member management function is responsible for authorizing and verifying members in the network, and the channel management function is responsible for the creation and closing of channels, consensus policies (roles, permissions, sorting, etc.), and the joining and exiting of nodes.

(3) Data layer: The main function of the data layer is to store key information that needs to be supervised in the process of data trading, and to ensure the security and privacy of trading information. The data layer includes an off-chain database and Hyperledger Fabric blockchain, in which the off-chain database mainly stores the data information verified by smart contracts and the mapping relationship information between the blockchain networks. The data on the blockchain are stored in the form of files. Blockchain distributed block data comprise information such as data blocks, hash functions, data digests, timestamps, Merkle trees, asymmetric encryption, digital signatures, public and private keys, chain structure, etc. The blockchain network has three types of trading supervision channels. Each channel maintains an independent ledger, and enterprise nodes choose to join different business channels according to business needs to achieve the isolation and protection of private information.

### 4.6. Formal Expression of Distributed Supervision Model

The distributed supervision model can be expressed by an 11-tuple {E,P,D,A,CM,T,TM,PF,VS,SV,TSC}:

(1) $E=\left\{e_{1},e_{2},\ldots ,e_{n}\right\}$ represents a non-empty finite set of enterprise users, in which each enterprise $e_{i}$ is a potential trading subject, that is, the demander, provider or server of data resources or services.

(2) $P=\left\{p_{1},p_{2},\ldots ,p_{n}\right\}$ represents the non-empty finite set of the platform. The platform $p_{i}$ acts as the intermediate platform of the data asset trading subjects. The demander needs to go through the platform to request data resources or services from the provider, and request data processing services from the server.

(3) $D=\left\{d_{1},d_{2},\ldots ,d_{n}\right\}$ represents a non-empty finite set of enterprise data resources, $d_{i}$ represents a certain type of dataset of an enterprise, and each dataset is uniquely affiliated to a certain user, that is, for $\forall d_{i}\in D$, $\exists e_{j}\in E$, satisfy the mapping $f:d_{i}\to e_{j}$.

(4) $A=\left\{a_{1},a_{2},\ldots ,a_{n}\right\}$ represents a non-empty finite set of enterprise data assets. Data assets are processed datasets and used for trading, which are the main form of enterprise data trading. A data asset can be the multi-type data of one enterprise, or the result of fusing of multi-type data of multiple enterprises. Let $CM=\left\{cm_{1},cm_{2},\ldots ,cm_{n}\right\}$ be the set of data capitalization methods, $A^{\prime }\subseteq A$, $CM^{\prime }\subseteq CM$, $a_{i}\in A$; then, the data asset $a_{i}=\left(A^{\prime },CM^{\prime }\right)$.

(5) $T=\left\{t_{1},t_{2},\ldots ,t_{n}\right\}$ represents a limited set of data asset trades. Each element represents a trade. The content of each trade may be different, and each trade may not necessarily make a profit, but the trading object can only be two (any trading subject and platform). Define $TM=\left\{tm_{1},tm_{2},\ldots ,tm_{n}\right\}$ as a non-empty finite set from using the data asset trading method. For $t_{i}\in T$, $P^{\prime }\subseteq P$, $E^{\prime }\subseteq E$, $tm_{i}\in TM$, let $PF=\left\{pf_{1},pf_{2},\ldots ,pf_{n}\right\},pf_{i}\geq 0$ be the income of this trading. Then, each trade can be expressed as $t_{i}=\left(E^{\prime },P^{\prime },tm_{i},pf_{i}\right)$.

(6) $VS=\left\{vs_{1},vs_{2},\ldots ,vs_{n}\right\}$ represents a non-empty finite set of data processing methods, mainly including the analysis, arrangement, calculation, editing and processing of data using various algorithms or models.

(7) $SV=\left\{sv_{1},sv_{2},\ldots ,sv_{n}\right\}$ represents a non-empty finite set of supervisors, including third-party supervisor, platform parties and alliance members other than trading. These supervisors conduct multi-party hierarchical supervision over the entire process of data asset trading.

(8) $TSC=\left\{tsc_{De-Pl-Su},tsc_{De-Pl-Pr-Su},tsc_{De-Pl-Se-Pr-Su}\right\}$ represents a finite set of trading supervision channels, which includes three types of trading supervision channels: $tsc_{De-Pl-Su}=\left\{tsc_{De-Pl-Su,1},tsc_{De-Pl-Su,2},\ldots ,tsc_{De-Pl-Su,n}\right\}$, $tsc_{De-Pl-Pr-Su}=\left\{tsc_{De-Pl-Pr-Su,1},tsc_{De-Pl-Pr-Su,2},\ldots ,tsc_{De-Pl-Pr-Su,n}\right\}$ and $tsc_{De-Pl-Se-Pr-Su}=\left\{tsc_{De-Pl-Se-Pr-Su,1},tsc_{De-Pl-Se-Pr-Su,2},\ldots ,tsc_{De-Pl-Se-Pr-Su,n}\right\}$. Let $SV^{\prime }\subseteq SV$; then, each trade can be expressed as $tsc_{i}=\left(t_{i},SV^{\prime }\right)$, where $t_{i}=\left(E^{\prime },P^{\prime },tm_{i},pf_{i}\right)$.

On the basis of analyzing the data trading process and its characteristics, the formal expression method of the main trading subjects and trading activities in the whole process of data trading are proposed from a rational point of view. This will be conducive to the modeling, analysis and optimization of the data trading process, and provide an effective way to achieve the dialectical thinking, a model-based expression method and intelligence in the data trading supervision.

## 5. Performance Testing and Analysis of Distributed Supervision Model

This paper conducted a series of experimental tests on a computer using i9-9900K@ 3.60 GHZ CPU and 16 GB RAM, mainly including a traceability function and on-chain function. By simulating the interaction of multiple users with the blockchain network, continuously adding records to the blockchain network, and the throughput performance, latency and success rate of the traceability function and on-chain function of the blockchain network under different request sending rates were tested.

### 5.1. Performance Testing and Analysis of the Traceability Function

#### 5.1.1. Query Time Testing and Analysis of the Traceability Function

The proposed distributed supervision model of data trading adopts the data hybrid storage method of “on-chain + off-chain”. Among them, the hash value of the data trading supervision information is stored on the blockchain, and the block number where it is stored is obtained. At the same time, the original data trading information and block number are stored in the off-chain database. The supervisor reads the data trading information and the corresponding block number from the off-chain database, and hashes the trading information to obtain the corresponding hash value. It is then compared with the corresponding hash value on the blockchain to determine whether the trading information has been tampered with.

In previous studies, many scholars adopted the storage model of storing all the original data on the blockchain. For example, reference [48] stores product processing, logistics and sales information all on the blockchain. This results in an extremely heavy load on the blockchain, extremely high operating costs, and extremely low query efficiency.

**Figure 9:**
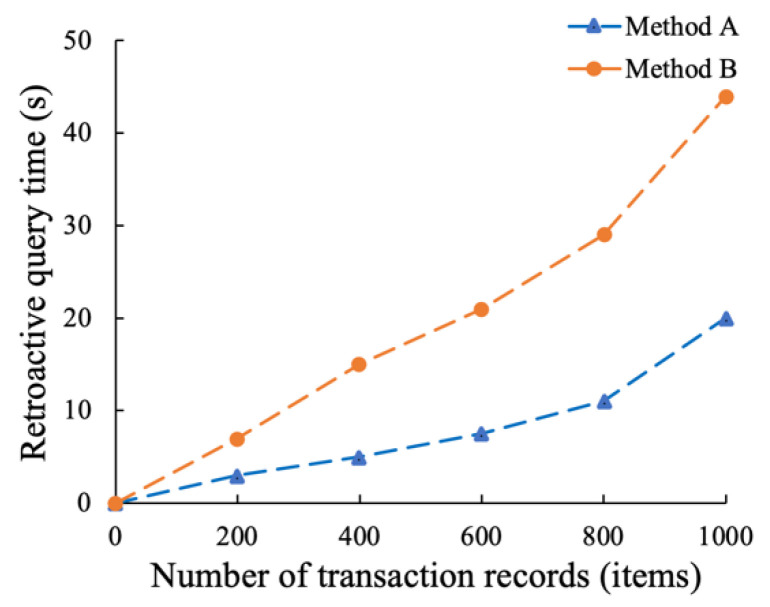
Time-consuming comparison of traceability for methods A and B.

The above two methods were compared and analyzed, and the same retrospective query operation was performed on them under the same conditions. The traceability times of these two methods on different numbers of transaction records were compared. The experimental results are shown in Figure 9, where method A is the proposed method, and method B is the method adopted in the literature [48].

As can be seen from Figure 9, as the number of transaction records gradually increases, the traceability times of both methods A and B increase linearly and gradually. However, the increasing slopes of the two methods are quite different. The traceability time in the whole range of method A is much lower than that of method B, and the time-consuming gap between the two methods tends to increase gradually with the increase in the number of transaction records. Compared with method B, the traceability time of the proposed method, method A, is lower than that of method B by an average of 58% when the number of transaction records is between 0 and 1000. The experimental results show that the hybrid storage mode of “on-chain + off-chain” in this paper has a higher operation and query efficiency.

#### 5.1.2. Throughput and Latency Testing of the Traceability Function

**Figure 10:**
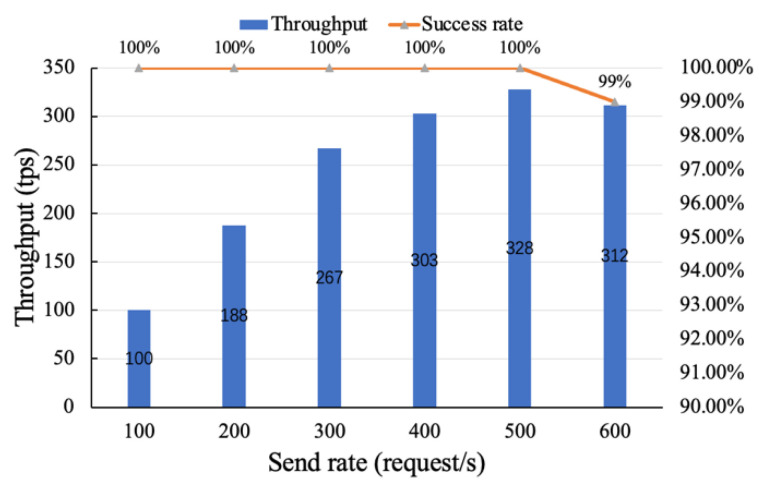
Throughput performance and transaction success rate of the traceability function.

The traceability function includes data resource traceability, data service traceability, etc. The throughput performance of the traceability function under different request sending rates was tested, and the experimental results are shown in Figure 10.

When the request sending rate is between 0 and 100, the throughput of the traceability function is approximately equal to the request sending rate, because the traceability function does not need to change the state on the chain through consensus. When the request sending rate is between 100 and 400, the throughput slowly increases and becomes stable. This is due to the increased number of requests and increased competition, resulting in a lower throughput growth rate. When the request sending rate exceeds 500, the throughput fluctuates around 320, indicating that the server I/O peaks at this time. This is due to excessive load at this time, which has reached its maximum throughput. Through the analysis of the experimental results, the throughput of the traceability function is about 328, which can meet the supervision of a certain number of the data asset trades of users. It can also be seen in Figure 10 that the overall transaction success rate stays above 99% when the throughput of the traceability function is at its peak.

**Figure 11:**
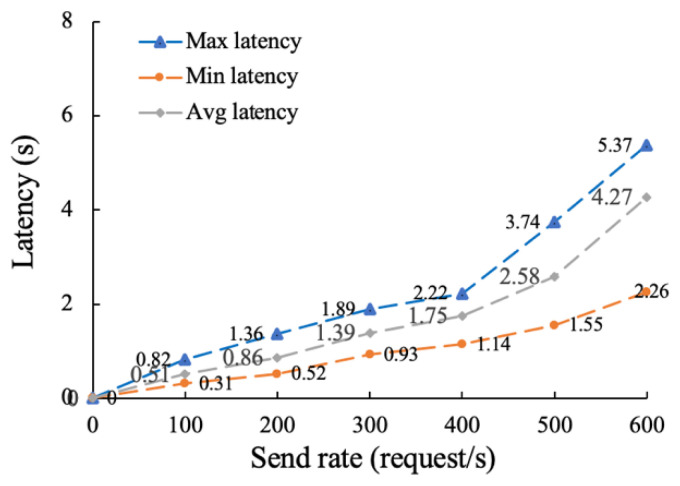
Latency testing of the traceability function.

Then, we conducted a total of six rounds of testing on the traceability function with the send rates set to 100, 200, 300, 400, 500 and 600. As can be seen from Figure 11, when the sending rate reaches about 500, the average transaction delay is about 2.58 s and the response speed is fast. When the sending rate is further increased to around 600, the response speed becomes slower, and the average transaction delay increases to 4.27 s. This is because competition between transactions is low when the number of requests is small, resulting in little latency.

### 5.2. Performance Testing and Analysis of the On-Chain Function

#### 5.2.1. Throughput Testing and Analysis of the On-Chain Function

**Figure 12:**
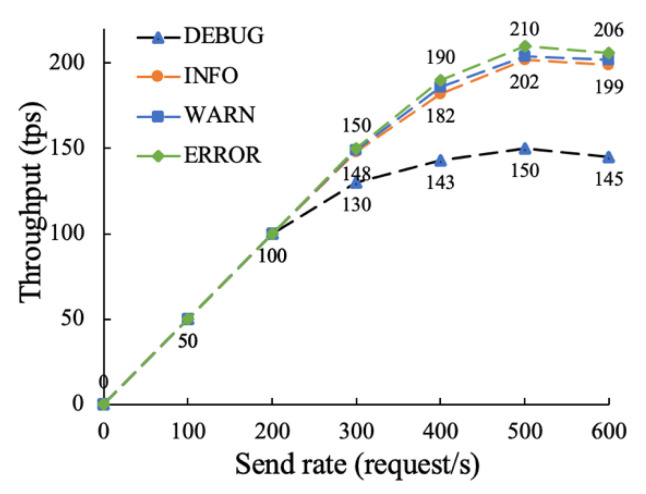
The impact of the Fabric system log level on the throughput performance of the on-chain function.

**Figure 13:**
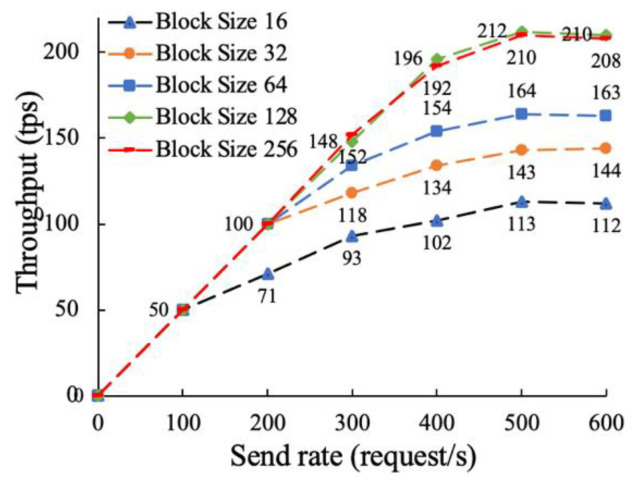
The effect of block size on the throughput performance of the on-chain function.

The on-chain function mainly includes data demand request, data resource release, data service result feedback, etc. The on-chain function is more complex and time-consuming than the traceability function. This paper conducts experimental tests by changing the log level and block size, and analyzes the impact of different parameters on throughput performance. The experimental parameter settings are shown in Table 3, and the results are shown in Figure 12 and Figure 13.

As can be seen from Figure 12, when the request sending rate is between 0 and 200, there is no significant difference in the throughput performance of each log level. When the request reach rate is between 200 and 400, the throughput of the DEBUG log level is gradually lower than that of the other log levels, because the lower the log level, the more detailed the output is, and it is easier to debug after a bug occurs. When the request reaching rate exceeds 500, each log level tends to be stable, and the remaining log levels are about 200 tps, far exceeding the 150 tps of the DEBUG log level. This is due to too many logs being outputted, which degrades the throughput performance of the model. In severe cases, the throughput performance can even degrade by several orders of magnitude. It can be seen from this that outputting too many logs may seriously degrade the throughput performance of the blockchain network, and the log levels on different working paths should be carefully adjusted in the actual environment.

It can be seen from Figure 13 that when the request sending rate is between 0 and 100, the block size only has little effect on the throughput performance. After the request sending rate gradually increased, the throughput performance began to improve as the block size increased. This is because the number of transactions a block can hold increases as the block size increases, thus increasing throughput. When the block size reaches 128 KB, the throughput performance hardly rises anymore and stabilizes around 212 tps, because the block transmission time will increase as the block size increases, thus reducing the throughput.

To sum up, it can be seen from Figure 12 and Figure 13 that the throughput performance of the on-chain function of this model is about 211 tps, and the parameters of the orderer node configuration file have a great influence on the performance of the model. In practical applications, appropriate parameters should be set according to the actual enterprise data asset trading scenario.

#### 5.2.2. Latency Testing and Analysis of the On-Chain Function

**Figure 14:**
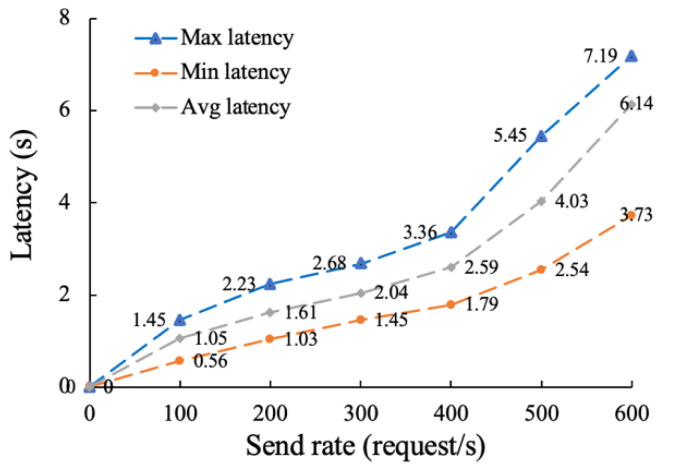
Latency testing of the on-chain function.

Similarly, we tested the latency of the on-chain function for six rounds, with request sending rates ranging from 100 to 600. The experimental results are shown in Figure 14.

As can be seen from Figure 14, when the sending rate reaches about 400, the average transaction delay is about 2.59 s, and the response speed is fast. When the sending rate is further increased to around 500, the average transaction latency increases to 4.03 s with a smaller drop in response speed. When the sending rate reaches around 600, the average transaction latency reaches 6.14 s, and the response speed drops greatly. This is because the system is already overloaded under high competition, resulting in increasing latency.

According to the above test results, the proposed distributed supervision mode of data trading based on blockchain has high throughput of traceability and on-chain function. When the sending rate is about 500, the average delay of the on-chain function is within 3 s, and the transaction success rate is 100%, which can meet the actual business needs of data trading supervision.

### 5.3. Security Analysis

The proposed data trading distributed supervision model is built based on Hyperledger Fabric alliance chain technology and has a high confidence computing environment with enterprise as the core. Moreover, the blockchain is non-tampering and traceable, and the events occurring in the blockchain are fully recorded in the log, which can prevent all entities from denying their actions during the trading. Considering the security, privacy and audit requirements of data asset trading, membership service provider (MSP) in Fabric is used to manage the on-chain licensing of enterprise members, and then the non-licensing chain is transformed into the licensing chain through public key infrastructure (PKI). In terms of transaction security, enterprise members who intend to trade data assets can not only apply for a long-term E-CERT, but also apply for a short-term T-CERT from the CA. Since the holder of T-CERT is only known to the transaction certification authority and the auditor, it can help the user to conduct anonymous transactions, and when the query information needs to be verified, the user’s identity can be authenticated by CA. In addition to certificates, two-way Transport Layer Security (TLS) authentication is enabled in Fabric, which not only enables clients to authenticate service nodes, but also enables service nodes to authenticate clients, ensuring communication security in the P2P environment of blockchain network.

In addition, the proposed model creates three types of supervision channels based on channel technology, namely De-Pl-Su, De-Pl-Pr-Su and De-Pl-Pr-Se-Su, to isolate different trading. This allows trading information to be shared only among the organizations involved in the data trading, while other organizations have no access to it, protecting the security of sensitive and private trading information. On this basis, the channel membership relationship can be managed by MSP, including: (1) authentication and identification of participants, (2) establishment of trust domains with the channel as the boundary, and (3) ability to identify the roles of participating entities. Finally, Signature Policy and ImplicitMeta Policies can be used to refine access control permissions according to the regulatory characteristics of different transaction businesses.

### 5.4. Discussion and Limitations

In conclusion, we test the proposed distributed supervision model in terms of traceability and on-chain performance, throughput and transaction delay. Compared with storing all trading information on the blockchain, the proposed “on-chain + off-chain” hybrid storage model has higher traceability and up-chain efficiency. When the number of transaction records is between 0 and 1000, the traceability time of the proposed method is still 58% lower than that of [48] on average. This is due to the fact that [48] stores all information in the blockchain, resulting in heavy load, high operating costs, and low query efficiency. By increasing the sending rate to 500 successively, it is found that the throughput performance of the traceability function is about 328 tps, which can meet the data asset transaction of a certain number of users. We also analyzed the throughput performance of the on-chain functions by adjusting the level of detail of the log output and the block size. It is found from experiments that outputting too much logs may seriously degrade on-chain performance. Therefore, log levels should be carefully adjusted for real-world scenarios. As the block size increase, the on-chain throughput increases, but when the block size reaches 128 KB, the on-chain throughput peaks at about 212 tps. This is because when the block size is too large, its transmission time will be long. Then, we then tested the latency and transaction success rate of the traceability and on-chain functions. In six rounds of tests with sending rates of 100 to 600, the success rates for traceability function remained above 99%. In the transaction delay test, when the sending rate is 500, the average transaction delay of on-chain and traceability function is about 4.03 s and 2.58 s, respectively, and the transaction success rate is 100%, which can meet the actual business requirements of data trading supervision.

Although the proposed model focuses on the supervision of the whole process of data transaction, its distributed supervision mode of “on-chain + off-chain” and the strategy of balancing the contradiction between privacy and supervision by using channel technology can also be extended to the supervision of e-commerce and other Internet business. In the construction of the proposed model, the on-chain and off-chain parallel operation mode is adopted, in which the blockchain stores only the key business information, while the off-chain is responsible for storing all the original trading information, so as to reduce the blockchain load and improve the operation efficiency. Finally, the channel technology in Fabric provides a new idea and method to balance the contradiction between privacy and regulation.

The security supervision of data trading based on blockchain technology studied in this paper focuses on the supervision of the entire process of data trading. Although blockchain technology can ensure the credibility and non-tampering of data after it is on the chain, the serial processing of data transactions by distributed consensus of blockchain network nodes cannot achieve the performance of traditional centralized supervision. Therefore, when the data trading supervision is applied on a large scale, it is faced with the problem of on-chain efficiency decline and capacity expansion caused by the continuous increase of supervision information, which is the topic direction of our further research in the future.

## 6. Conclusions

In order to enhance the supervision ability of data trading, adopt the strategy of A-V-T (access–verification–traceability), and according to the principle of minimization, a distributed supervision model of data trading based on Fabric multi-channel technology is designed to realize the collaborative supervision before the event, at present and after the event over the whole process of data trading. Firstly, through a comprehensive analysis of the data trading business process and its supervision characteristics, five typical links of data trading access and online, supply and demand matching, trading execution, trading settlement and data services were abstracted. On this basis, a classification table of key supervision information in each link of the whole process was proposed. Secondly, based on the Fabric multi-channel technology, three types of supervision channels, De-Pl-Su, De-Pl-Pr-Su and De-Pl-Se-Pr-Su, were constructed, and corresponding types of actual supervision channels were created according to the requirements of data trading business types. Among them, each data trading supervision channel operates independently and in parallel, and the natural isolation of the channel was used to solve the protection of data privacy among multiple enterprise entities in supervision. The supervisors, which are composed of supervisory agencies, the platform and representatives of alliance members, join each data trading supervision channel to realize pre-entry verification supervision, the in-process verification and comparison supervision, and the post-event retrospective audit supervision. Subsequently, a supervision smart contract was created to realize differentiated supervision for multi-party hierarchical collaborative supervision, and through the system channel to realize the necessary information exchange between each trading supervision channel, to effectively manage and control each data trading supervision channel, and to solve the problem of permanent traceability afterwards. Finally, the “on-chain + off-chain” hybrid storage methods for supervision information and the on-chain hierarchical structure of the data trading supervision model are proposed, which can reduce the load of blockchain and improve the efficiency of operation and query.

In this paper, we test and analyze the throughput performance, transaction success rate and latency of the traceability and on-chain functions in the proposed model by simulating multi-node deployment. The experimental results show that the proposed distributed supervision model of data trading based on blockchain multi-channel is feasible and effective, which can effectively solve the two main problems of privacy and supervision, denial and traceability, and improve the quality and efficiency of data trading. The distributed supervision mode of “on-chain + off-chain” and the strategy of balancing the contradiction between privacy and regulation using channel technology proposed in this paper can also be extended to the transaction or exchange regulation of other businesses.

## Figures and Tables

**Table 1:** Key information of each link in the data trading process.

Data Trading Links	Key Information	
	Public Supervision Information	Private Supervision Information
data access	data source information, data type, data format, data size, update time, preprocessing information.	data quality, sales price, owner information.
supply and demand match	functional or target requirements, standards or specifications to be met, purchase quantity, delivery time, dataset size, dataset description.	purchase price, sale price, delivery method, data storage address.
trading implementation	purchase method, delivery time, dataset name, data online time, abstract of delivery content.	delivery method, selling company.
trading settlement	order number, payment method, payment amount, payment time.	platform handling fee, trading time, trading price.
data service	server information, data service requirements, data service type, completion time.	processing price, result delivery method.

**Table 2:** Symbol table.

Symbol	Description
EnterPeer	The enterprise node.
SupPeer	The supervision node.
$Key_{supchan}$	Supervision channel authorization file.
$Key_{oriinfo}$	Off-chain database authorization file.
PriSupInfo	Privacy trading supervision information.
PubSupInfo	Public trading supervision information.
TradingName	The name of the data trading.
BlockNum	It represents the location of the hash value of the data trading supervision information in the blockchain.
txID	Transaction hash.
Veriresult	Verification results of data trading information traceability process.
Veripass	The on-chain and off-chain hash values are the same, and the verification passes.
Verifail	Verification failed because the hash values on-chain and off-chain are inconsistent.

**Table 3:** Experimental parameter settings.

Number	Logging Level	Block Size/KB
1	DEBUG/INFO/WARN/ERROR	64
2	INFO	16/32/64/128/256

## Data Availability

Not applicable.
